# Risk of stroke and retinopathy during GLP-1 receptor agonist cardiovascular outcome trials: An eight RCTs meta-analysis

**DOI:** 10.3389/fendo.2022.1007980

**Published:** 2022-12-05

**Authors:** Jinjing Wei, Bing Yang, Ruxin Wang, Haowen Ye, Ying Wang, Lihong Wang, Xiaofang Zhang

**Affiliations:** ^1^ Department of Endocrinology and Metabolism, First Affiliated Hospital of Jinan University, Guangzhou, China; ^2^ The Guangzhou Key Laboratory of Basic and Translational Research on Chronic Diseases, The First Affiliated Hospital, Jinan University, Guangzhou, China; ^3^ Department Clinical Experimental Center, First Affiliated Hospital of Jinan University, Guangzhou, China

**Keywords:** ischemic stroke, stroke, T2DM, GLP-1RA, CVOTs (cardiovascular outcome trials)

## Abstract

**Purpose:**

To explore the risk of stroke (including ischemic and hemorrhagic stroke) in type 2 diabetes mellitus treated with glucagon-like peptide 1 receptor agonist (GLP-1RA) medication according to data from the Cardiovascular Outcome Trials(CVOT).

**Methods:**

Randomized controlled trials (RCT) on GLP-1RA therapy and cardiovascular outcomes in type 2 diabetics published in full-text journal databases such as Medline (*via* PubMed), Embase, Clinical Trials.gov, and the Cochrane Library from establishment to May 1, 2022 were searched. We assess the quality of individual studies by using the Cochrane risk of bias algorithm. RevMan 5.4.1 software was use for calculating meta- analysis.

**Results:**

A total of 60,081 randomized participants were included in the data of these 8 GLP-1RA cardiovascular outcomes trials. Pooled analysis reported statistically significant effect on total stroke risk[RR=0.83, 95%CI(0.73, 0.95), *p*=0.005], and its subtypes such as ischemic Stroke [RR=0.83, 95%CI(0.73, 0.95), *p*=0.008] from treatment with GLP-1RA versus placebo, and have no significant effect on the risk of hemorrhagic stroke[RR=0.83, 95%CI(0.57, 1.20), *p*=0.31] and retinopathy [RR=1.54, 95%CI(0.74, 3.23), *p*=0.25]

**Conclusion:**

GLP-1RA significantly reduces the risk of ischemic stroke in type 2 diabetics with cardiovascular risk factors.

## 1 Introduction

IDF(International Diabetes Federation)Releases New Global Diabetes Map 2021( http://www.diabetesatlas.org/), the data show that 537 million cases of adult diabetes worldwide by 2021. A variety of complications can arise from the progression of diabetes, and cardiovascular complications are the main cause of death in diabetics, for example, heart attack, brain attack, heart failure, malignant arrhythmia, etc. Stroke is a brain disorder that is attributed to a dramatic blood vessel rupturing or blocking within the brain, thereby preventing the flow of blood to the brain. Nearly a population of 15 million encounter stroke each year in the globe, with 33% caused to be permanently disable, and 40% caused to die (1, 2). Diabetes is now widely recognized nationally and internationally as a major and independent risk factor for stroke morbidity and mortality. A study in 2022 《the European Journal of Preventive Cardiology》 showed that type 2 diabetics are at high or very high risk of fatal myocardial infarction (MI) or stroke (3). Recent data from a study conducted by Wang Congjun at Beijing Tiantan Hospital in China showed that 33.4% of 833,000 acute ischemic stroke patients have combined diabetes. As the pathological basis of most ischemic strokes and some hemorrhagic strokes is atherosclerosis, While diabetes can precipitate or exacerbate the development of atherosclerotic lesions, Thus, it is so important to pay attention to the atherosclerosis-protective effect when developing individualized glucose-lowering regimens for type 2 diabetes mellitus (T2DM) that reduce the risk of stroke. GLP-1RA is a new type of glucose-lowering drug that decreases the risk of hypoglycemia by stimulating insulin secretion and lowering glucagon secretion, delaying gastric emptying, and reducing appetite to lowers HbA1c, modestly improves blood lipids and body weight (4). A meta-analysis study showed that GLP-1-based therapies appear to provide beneficial effects against atherosclerosis (5). A pooled analysis reported GLP-1RA no significant effect on atherosclerotic MACE (RR 0.91, 95% CI 0.84–1.00, p=0.05) (6). International attention is mainly focused on the cardiac and renal outcomes of GLP-1RA clinical trials, there are other good meta-analyses and umbrella reviews analyzing MACE events (including nonfatal stroke) (7–11). But less attention has been paid to the question of whether GLP-1RA treatment can reduce the risk of subtype of stroke in patients with T2DM. On the other hand, the 2013 American Heart Association (AHA)/American Stroke Association (ASA) Expert Consensus on “A New Definition of Stroke for the 21st Century” (the “Updated Consensus”) concluded that, Central nervous system (CNS) infarction is defined as “ischemic cell death in the brain, spinal cord, or retina based on pathology, imaging, other objective evidence, and/or clinical evidence of ischemia” (12). Retinal ischemia secondary to central retinal artery occlusion meets the definition of acute ischemic stroke. Therefore we aimed to perform a meta-analysis of adverse event outcomes of stroke and retinopathy in the GLP-1RA large-scale CVOT clinical trial (RCT).

## 2 Materials and methods

### 2.1 Search strategy

This meta-analysis was aligned with the Preferred Reporting Items for Systematic Reviews and Meta-Analyses (PRISMA) guidelines (www.prisma-statement.org), the protocol was registered in The International Prospective Register of Systematic Reviews (PROSPERO) (www.crd.york.ac.uk/prospero) under (CRD42022337176). The English and Chinese literature on GLP-1RA associated with cardiovascular outcomes published since the establishment of the database until May 1, 2022 was searched manually by computer in MEDLINE (*via* PubMed), Embase, Clinical Trials.gov, and Cochrane Library databases. Search terms: “Glucagon-like peptide-1 receptor agonist”, “Cardiovascular”, “ cardiac failure”, “lixisenatide”, “exenatide”, “liraglutide “, “Semaglutide”, “Albiglutide”, “Dulaglutide”, “Efpeglenatide”, “placebo”, “Clinical Trials”.

### 2.2 Inclusion criteria and exclusion criteria

Inclusion criteria: (1) Randomized, double-blind, parallel-group, multicenter study of clinical trials; (2) Type 2 diabetes mellitus who are at high cardiovascular risk (including but not limited to obesity, metabolic syndrome, insulin resistance, hypertension, dyslipidemia, etc.) are the primary study subjects; (3) At least 1000 people in the test and control groups, and at least 3000 people in total; (4)Intervention with GLP-1 receptor agonist and control with placebo; (5)Data on ischemic stroke, hemorrhagic stroke and retinal arteriopathy must be available in all trials for adverse events; (6)A more complete table of baseline patient characteristics is available; (7)Published English literature up to May 1, 2022.

Exclusion criteria: (1) Reviews, reports, and conference proceedings on GLP-1RA and cardiac arrhythmias; (2) Studies with inaccessible full text or incomplete data; (3) Repeatedly published or repeatedly included studies or studies with similar information; (4) Clinical trials that included type 1 diabetic patients.

### 2.3 Main and secondary results

#### 2.3.1 Primary outcome

Total stroke events and major stroke types (including ischemic stroke and hemorrhagic stroke).

#### 2.3.2 Secondary outcomes

Retinopathy (Retinopathy means disease of the retina. There are several types of retinopathy but we only include hemorrhagic and ischemic related retinopathy)

### 2.4 Literature screening, data extraction and quality evaluation

Randomized controlled trials comparing GLP-1RA with placebo in T2DM at high cardiovascular risk were included. Outcomes of interest included stroke events and retinopathy. First, titles and abstracts were screened to assess their potential eligibility for inclusion, and then full-text checks were applied to determine final eligibility. The following information was collected using a predefined data extraction form: study information (trial name, sample size, drug name), patient characteristics (age, gender, baseline status), therapy information (regimen, dose) and outcome data (number of events per outcome). All outcomes of interest were dichotomous, first preferentially extracting data from ClinicalTrials.gov and secondarily selecting data from the original trial publication or secondary analysis of the same trial. The Cochrane Risk of Bias Tool was used to assess the quality of included studies (13). Bias was assessed in seven ways: selection bias (including random sequence generation, allocation concealment), implementation bias (whether subjects and trial personnel were blinded), measurement bias (whether outcome assessors were blinded), follow-up bias (whether outcome data were complete), reporting bias (whether study outcomes were selectively reported), and other bias (whether there were other sources of bias). The assessment criteria levels are classified as high, low or unclear. If one item was judged to be high, the overall risk of bias was judged to be high, and if all items were judged to be low, the risk of bias was judged to be low, otherwise it was unclear.

### 2.5 Statistical methods

Data were analyzed with RevMan 5.4.1, and effect analysis statistics were expressed as RR and 95% CI, *p <*0.05 being a statistically significant difference. Heterogeneity analysis among groups between studies was executed using the χ^2^ test, and the results are presented as *I^2^*. Fixed-effects models were used for analysis if there was homogeneity among studies (*p >*0.05 or *I^2^ ≤* 50%), and random-effects models were used for analysis if there was heterogeneity among studies (*p ≤* 0.05 and *I^2^* >50%). Sources of heterogeneity can be searched for by sensitivity analysis and subgroup analysis when large heterogeneity exists. Due to the number of trials being less than 10, publication bias was not evaluated.

## 3 Result

### 3.1 Procedure and outcomes of included literature

**Figure 1:**
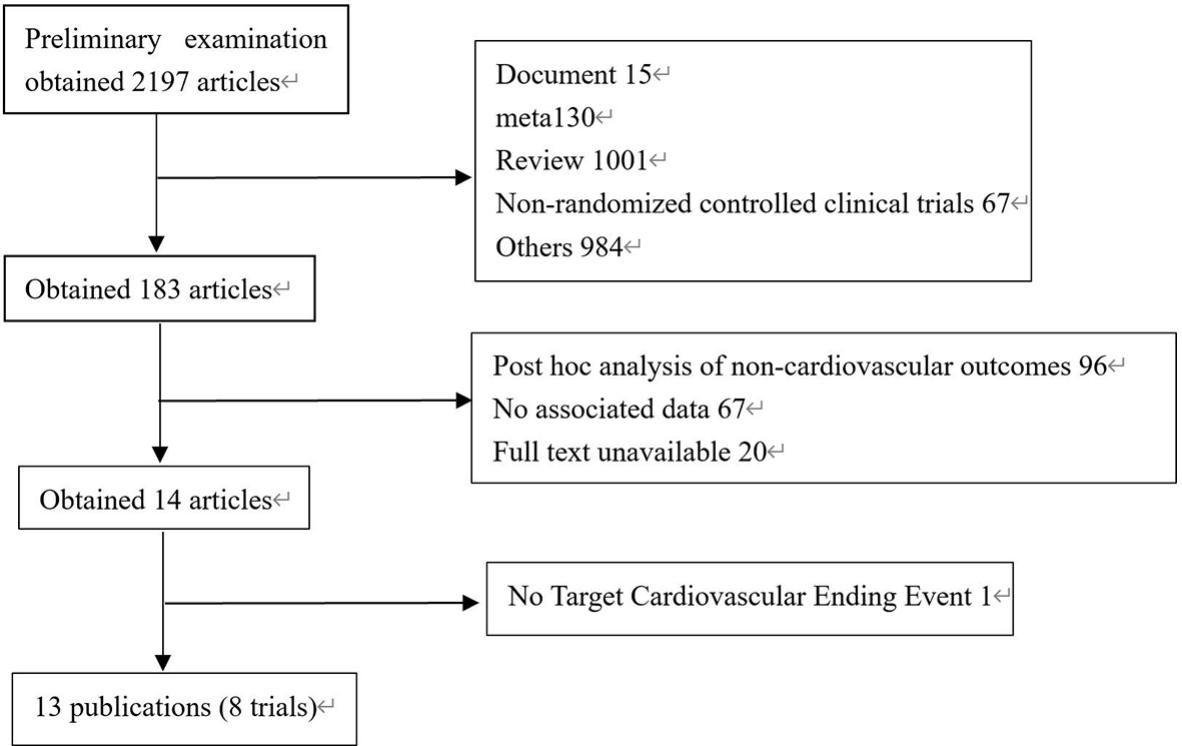
Process of studies’ selection.

2008 literatures were initially screened in the database according to keywords, and 176 were obtained after de-duplication and exclusion of review literature, irrelevant literature, incomplete data, unreported cardiovascular events, unspecified results, and incorrect study type, and then 8 clinical trials with a total of 68,001 patients were finally included after the screening process was repeated by two investigators again (**Figure 1**). The included reports, in chronological order, were ELIXA (14)、LEADER (15)、SUSTAIN-6 (16)、EXSCEL (17)、Harmony Outlets (18)、REWIND (11)、PIONEER 6 (10) and AMPLITUDE-O (9). Key trial and patient characteristics at baseline examination are shown in **Table 1**. All trials were of considerable size (>3000 patients). Of the 8 trials, ELIXA recruits patients with a recent acute coronary syndrome, and the study populations of the other 7 trials indicated in their inclusion criteria that they primarily included patients with stable cardiovascular disease or cardiovascular risk factors (19). In all eight trials, local investigators were encouraged to manage participants according to local guidelines.

**Table 1:** Baseline characteristics of the included studies.

	ELIXA(2015)	LEADER(2016)	SUSTAIN-6(2016)	EXSCEL(2017)	Harmony Outcomes (2018)	REWIND(2019)	PIONEER 6(2019)	AMPLITUDE-O(2020)
**Drug**	Lixisenatide	Liraglutide	Semaglutide	Exenatide	Albiglutide	Dulaglutide	Semaglutide	Efpeglenatide
**paticipants**	6068	9341	3297	14752	9463	9901	3183	4076
**Usage**	20μg sc qd	1.8mg sc qd	0.5 or 1mg sc qw	2mg sc qw	30 or 50mg qw	1.5mg sc qw	14mg po qd	4 or 6mg sc qw
**mean follow up (years)**	2.1	3.8	3.1	3.2	1.6	5.4	1.3	1.8
**Mean age(years)**	60.3 ± 7	64.3 ± 7	64.6 ± 7	61.9 ± 9	64.1 ± 7	66.2 ± 7	66.0 ± 7	64.5 ± 7
**Male n (%)**	4207(69.3%)	6003(64.3)	2002(60.1%)	9149(62%)	6569(69.4%)	5312(53.7%)	2176(68.4%)	2732(67%)
**BMI(kg/m^2^)**	30.2 ± 6.2	32.5 ± 6.3	32.8 ± 6.2	31.8 ± 6.4	32.3 ± 5.9	32.3 ± 5.7	32.3 ± 6.5	32.7 ± 6.3
**Established CVD n(%)**	6068(100%)	7598(81%)	2735(83%)	10782(73%)	9463(100%)	3109(31.4%)	2695(84.7%)	3650(89.6%)
**Duration of diabetes(years)**	9.2 ± 8.1	12.8 ± 8.0	13.9 ± 8.1	13.1 ± 8.3	14.2 ± 8.6	10.5 ± 7.2	14.9 ± 8.5	15.4 ± 8.2
**Mean HbA1c (%)**	7.7 ± 1.5	8.7 ± 1.6	8.7 ± 1.5	8.1 ± 1.0	8.7 ± 1.5	7.3 ± 1.1	8.2 ± 1.6	8.9 ± 1.5

Data are mean ± SD, unless otherwise noted.

### 3.2 Analysis of stroke types

#### 3.2.1 Total stroke events

All 8 included studies can be used to analyze the effect of GLP-1RA on total stroke. No significant heterogeneity among studies(*I^2 =^* 0%, *p* =0.71), so the fixed-effects model was used to combine the effect sizes. Pooled analysis reported statistically significant effect on total stroke outcomes from treatment with GLP-1RA versus placebo [RR=0.83, 95%CI(0.73, 0.95), *p*=0.005], showed that the risk of total stroke was about 17% lower in the GLP-1RA treatment group than the placebo group.

#### 3.2.2 Ischemic stroke events

All 8 included studies can be used to analyze the effect of GLP-1RA on ischemic stroke. No significant heterogeneity among studies(*I^2 =^* 0%, *p* =0.87), so the fixed-effects model was used to combine the effect sizes. Pooled analysis reported statistically significant effect on ischemic stroke outcomes from treatment with GLP-1RA versus placebo [RR=0.83, 95%CI(0.73, 0.95), *p*=0.008], showed that the risk of ischemic stroke was about 17% lower in the GLP-1RA treatment group than the placebo group.

#### 3.2.3 Hemorrhagic stroke events

**Figure 2:**
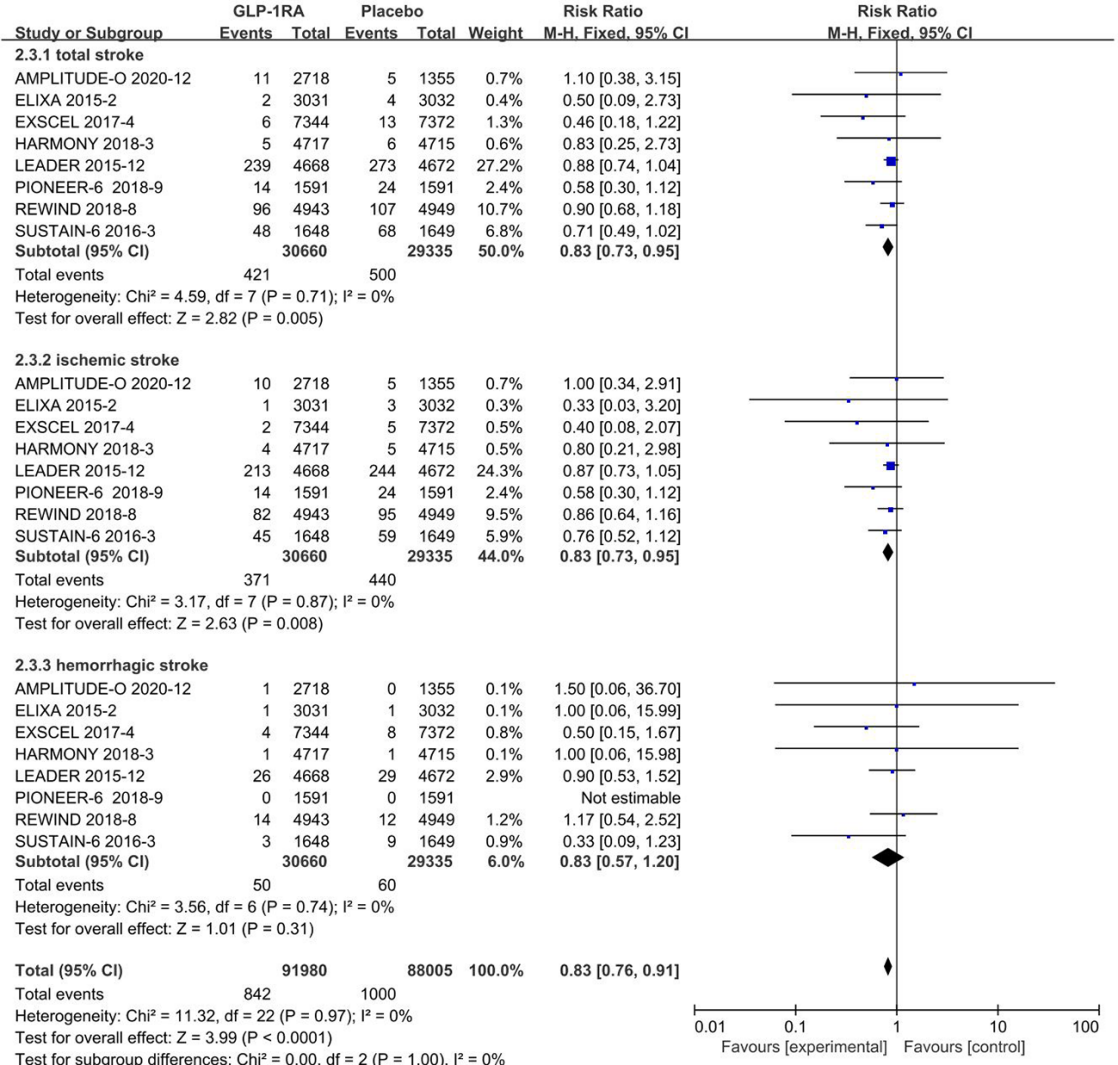
Forest map of stroke risk.

The eight studies collected for mate-analysis of the effect of GLP-1RA on hemorrhagic stroke are listed in **Figure 2**. No significant heterogeneity among studies (*I^2 =^* 0%,*p* =0.74), so the fixed-effects model was used to combine the effect sizes. Pooled analysis reported no significant effect on hemorrhagic stroke outcomes from treatment with GLP-1RA versus placebo [RR=0.83, 95%CI(0.57, 1.20), *p*=0.31].

### 3.4 Analysis of secondary outcomes

#### 3.4.1 Retinal arteriopathy

**Figure 3:**
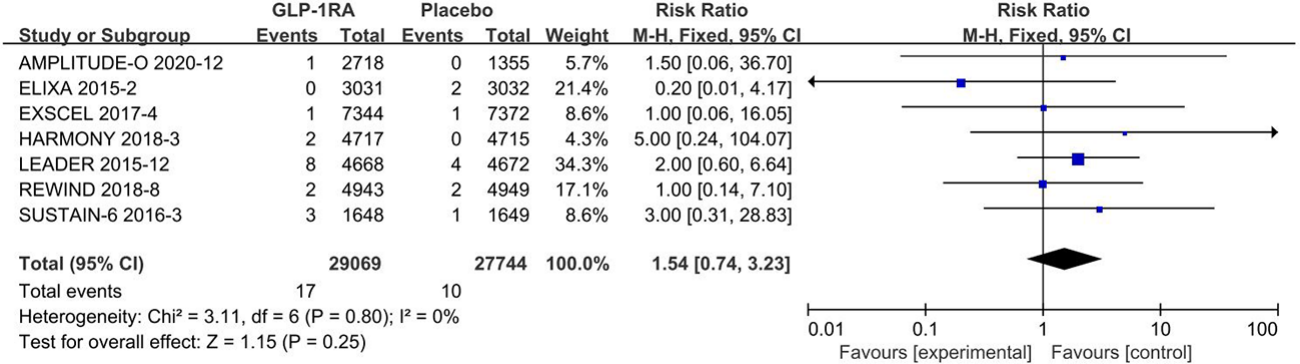
Retinal arteriopathy risk forest map.

All 8 included studies can be used to analyze the effect of GLP-1RA on retinal arteriopathy are listed in **Figure 3**. No significant heterogeneity among studies(*I^2 =^* 0%, *p*=0.80), so the fixed-effects model was used to combine the effect sizes. Pooled analysis reported no significant effect on retinal arteriopathy outcomes from treatment with GLP-1RA versus placebo [RR=1.54, 95%CI(0.74, 3.23), *p*=0.25].

## 4 Discussion

The results of eight RCTs on CVOT outcomes of GLP-1RA in type 2 diabetic patients, which we included, showed that GLP-1RA treatment significantly reduced the risk of total stroke (~17%) and ischemic stroke (~17%) in type 2 diabetic patients, suggesting that GLP-1RA may have some protective effect in patients with cerebrovascular stenosis. Similar studies have been done in the past, A 2022 retrospective cohort study reported longer use and higher dose of GLP-1 RAs were associated with a decreased risk of hospitalization for ischemic stroke among Asian patients with T2DM who did not have established atherosclerotic cardiovascular diseases, but who did have dyslipidemia or hypertension (20). A net meta-analysis in 2021 showed GLP-1RA versus placebo Our findings indicate that GLP-1RA reduce the risk of stroke (OR 0.87, 95% CI 0.77 to 0.98; high-certainty evidence) (7). Another study in 2021 comparing the efficacy and safety of SGLT2I, GLP-1RA and DPP4i found that only GLP-1RA was associated with a lower risk of stroke compared with placebo (RR 0.85, 95% CI 0.76, 0.94) (21). Some trials showed that there was a significant (*p* = 0.012) 9% risk reduction of non-fatal stroke associated with the use of newer glucose-lowering drugs, which was largely driven by the 16% reduction associated with the use of GLP-1RA, with no significant heterogeneity (*I^2^* = 21.3%, *p* = 0.206) and no evidence of publication bias (Egger test, *p* = 0.233) (22). Besides, a clear significant benefit on non-fatal stroke were showed on SUSTAIN-6 and REWIND for GLP-1RA and SCORED for SGLT-2i (22).This suggests that perhaps GLP-1RAs with longer half-lives appear to be beneficial for preventing MACE generally and possibly reducing the risk of stroke (23). Overall, the novelty of our study vs previous studies include the following: First, previous studies have focused more on MACE outcomes in patients with T2DM treated with GLP-1RA, capturing the risk of non-fatal stroke as a whole——as for a meta-analysis of the eight trials was already reported (24), while our study focuses on the risk of subtype of stroke after GLP-1RA treatment in T2DM. Second, previous studies have focused on cross-sectional comparisons of the cardiac and renal outcomes of various hypoglycemic drugs, the meaning is to compare the differences in safety and efficacy of different types of hypoglycemic drugs in application. We aimed to analyze the subtype risk of GLP-1RA in T2DM, to explore whether GLP-1RA may have a protective effect on diabetic patients with different high-risk cerebrovascular factors, and to provide a theoretical basis for further studies.

An interesting and important finding that administration of GLP-1Ras did not lower the risk for hemorrhagic stroke with statistical significance, but the RR was equivalent to that for ischemic stroke. It could be just due to the number of events and power. Although this meta-analysis showed that the RRs for hemorrhagic stroke were no statistical significance, the overall absolute risk increase should be of concern (50 events in 29069 patients treated with a GLP-1RA), because of its high disability and lethality rates.

Retinopathy has been reported as an serious adverse event in the published reports and/or the supplemental materials of the eight randomized clinical trials of GLP-1 Ras. We collected the data in the public disclosure one by one, and to maintain homogeneity, we only include hemorrhagic and ischemic related retinopathy, and excluded retinopathy due to other causes (e.g., glaucoma, cataract, etc.). However, to our knowledge, retinopathy events have seldom been reported. only 17 events in 29069 patients treated with a GLP-1RA eligible for this systematic review clearly reported retinopathy events, underreporting cannot be ruled out.

The main mechanisms of cardiovascular protection by GLP-1RA are currently explored as follows: anti-inflammatory, anti-atherosclerosis, reduction of reactive oxygen species production and anti-oxidative stress, reduction of thrombotic events, etc. Ex-4 inhibits oxidized LDL-induced macrophage foam cell formation by downregulating several inflammatory and adhesion molecules in monocytes and macrophages to suppress their accumulation in the arterial wall (25). Evidence from preclinical studies suggests that liraglutide was shown to inhibit TNF-α-induced oxidative stress and inflammation in endothelial cells through calcium and AMPK-dependent mechanisms (26, 27), and reduce the occurrence and progression of atherosclerotic plaque formation at an early stage and enhance plaque stability (28). Furthermore, GLP-1RA also reduces the inflammatory cytokines TNF-α, IL-1β and IL-6 (29), GLP-1RAs are also proposed to have antioxidant and neuroprotective effects by upregulating vascular endothelial growth factor production (30) and reducing proinflammatory cytokine production (31). Oeseburg et al. showed that GLP-1RA can prevent ROS through induced expression of antioxidant genes downstream of protein kinase A (PKA) and cAMP response element-binding (CREB) protein (32). Shi et al. suggested that liraglutide could protect cells from glucotoxic damage by inhibiting ERK1/2 and PI3K/Akt signaling pathways through GLP-1RA (33). a single injection of Ex-4 inhibited thrombus growth in normoglycemic and hyperglycemic mice in an *in vivo* laser injury model of arterial thrombosis (34). In a real-world study, liraglutide was found to significantly reduce cIMT (surrogate marker of subclinical atherosclerosis) in subjects with metabolic syndrome (MetS) during 18 months of follow-up, with a statistically significant reduction after only 6 months of treatment (35). More directly, intracerebroventricular administration of liraglutide reduced the cerebral infarct volume in rats with ischaemia–reperfusion injury (23, 30). These data suggest that GLP-1RAs have anti-atherosclerotic or vasculoprotective properties, which may be the main mechanism of their beneficial effect on stroke prevention (23).

Evidence from animal studies suggests that GLP-1-based therapies may be used as cardio- or neuroprotectants, suggests that GLP-1RAs and Dipeptidyl Peptidase-4 inhibitors (DPP-4is) may provide neuroprotection (36). However, a review suggests that DPP4i do not reduce any risk of efficacy outcomes, while moderate-certainty evidence likely supports the use of GLP-1RA to reduce fatal and non-fatal stroke (7). Moreover, research shows that GLP-1RA is the only drug class that reduces the risk of stroke among the various types of new hypoglycemic drugs (21). In terms of the molecular mechanisms, GLP-1 peptides shown more interesting actions, may cause GLUT4 upregulation in SHRs due to GLP-1 action (37). Glut4 mRNA expression and sarcolemmal translocation were also increased after GLP-1 stimulation in high-fatty acid incubated cardiomyocytes. PI3K/Akt and AMPKα were involved in this response (38). Low-level expression of Glut4 was observed in motor nuclei of spinal cord, nuclei of medulla oblongata, cerebellar nuclei and Purkinje cell layer, basal ganglia, neocortex, olfactory bulb, hypothalamus, and hippocampus in rodents (39–42),GLUT4/Glut4 in brain is supposed to be involved in provision of metabolic energy for firing neurons (43), and is supposed to be also involved in hypothalamic regulation of food intake, energy expenditure, and whole-body glucohomeostasis (44). However, data from clinical trials only report therapeutic efficacy for GLP-1RAs (36). Thus, GLP-1RA administration is the most promising treatment to pursue for patients at risk of stroke or immediately after stroke (36).

Our study also has limitations. First, these studies were not specifically designed to evaluate the risk of gallbladder or biliary diseases associated with GLP-1 Ras treatment, further validation could be done in future by designing large clinical studies with stroke as an endpoint. Second, we were unable to use patient-level data to evaluate outcomes, which limited our capability to further explore any subgroups of interest, Therefore, the findings of the subgroup analysis have to be interpreted cautiously and further investigations are needed to assess whether GLP-1RA may reduce the incidence of stroke in T2DM with multiple comorbidities. Third, the small number of events in subgroups may have allowed for underpowered subgroup analyses. Fourth, we included RCTs with a total of at least 3000 participants to limit bias due to small base size, which may have missed some of the positive or negative data. Fifth, no further animal or cellular experiments were done in this study to verify. The above limitations may weaken the persuasiveness of our study, but the majority of eligible studies selected by 2 different investigators based on strict inclusion criteria were of high quality through a comprehensive literature search of 4 databases. Thus, we believe the conclusions drawn from this meta-analysis are reasonable.

## 5 Conclusion

Overall, our study showed that GLP-1RA significantly reduced the risk of total stroke (about 27%) and ischemic stroke (about 27%) in T2DM, with statistically significant differences, and showed a neutral effect of GLP-1RA on the risk of hemorrhagic stroke and retinopathy.

## Data availability statement

The original contributions presented in the study are included in the article/**Supplementary Material**. Further inquiries can be directed to the corresponding authors.

## Author contributions

JW designed the research, JW and BY contributed to the literature database search, data collection, data extraction, data analysis, and writing of the manuscript. JW, BY, RW, HY and YW participated in the discussion. JW, BY, RW, XZ and LW reviewed and revised this article. All authors contributed to the article and approved the submitted version.

## Funding

This study was funded by Science and Technology Projects in Guangzhou (no. 20220102 0081), Talent introduction funding project of the First Affiliated Hospital of Jinan University (no. 808026) and Basic Scientific Research Project of central Universities of Jinan University (no. 21622301).

## Conflict of interest

The authors declare that the research was conducted in the absence of any commercial or financial relationships that could be construed as a potential conflict of interest.

## Publisher’s note

All claims expressed in this article are solely those of the authors and do not necessarily represent those of their affiliated organizations, or those of the publisher, the editors and the reviewers. Any product that may be evaluated in this article, or claim that may be made by its manufacturer, is not guaranteed or endorsed by the publisher.
