# Supplementary material for: Risk of stroke and retinopathy during GLP-1 receptor agonist cardiovascular outcome trials: An eight RCTs meta-analysis
Source: Front Endocrinol (Lausanne). 2022 Dec 5;13:1007980. doi: 10.3389/fendo.2022.1007980 (PMC9760859; doi:10.3389/fendo.2022.1007980)
Supplement: Supplementary file 1 [file Presentation_1.pdf]

The primary and secondary cardiovascular outcomes pre-selected in 8 studies

1. ELIXA：

Primary Outcome Measures :

Time to First Occurence of Primary CV Event: CV Death, Non-Fatal MI, Non-Fatal Stroke or Hospitalization for Unstable Angina [Time Frame: From randomization up to the end of study (median follow-up of 25 months)]

Secondary Outcome Measures :

Time to First Occurence of CV Event: CV Death, Non-Fatal MI, Non-Fatal Stroke, Hospitalization for Unstable Angina or Hospitalization For Heart Failure [Time Frame: From randomization up to the end of study (median follow-up of 25 months)]

Time to First Occurence of CV Event: CV Death, Non-Fatal MI, Non-Fatal Stroke, Hospitalization for Unstable Angina, Hospitalization For Heart Failure or Coronary Revascularization Procedure [Time Frame: From randomization up to the end of study (median follow-up of 25 months)]

Percent Change From Baseline in the Urinary Albumin/Creatinine Ratio (UACR) at Week 108 [Time Frame: Baseline to Week 108 (LOCF)]

2、LEADER

Primary Outcome Measures :

Time From Randomisation to First Occurrence of Cardiovascular Death, Non-fatal Myocardial Infarction, or Non-fatal Stroke (a Composite Cardiovascular Outcome) [Time Frame: from randomisation (visit 3; month 0) to last contact (visit 16; up to month 60+30 days)]

Secondary Outcome Measures :

Time From Rand. to First Occurrence of an Expanded Composite Cardiovascular Outcome Defined as Either Cardiovascular Death, Non-fatal Myocardial Infarction, Non-fatal Stroke, Revascularisation, Hospitalisation for Unstable Angina or for Heart Failure. [Time Frame: from randomisation (visit 3; month 0) to last contact (visit 16; up to month 60+30 days)]

Time From Randomisation to All Cause Death [Time Frame: from randomisation (visit 3; month 0) to last contact (visit 16; up to month 60+30 days)]

Time From Randomisation to Each Individual Component of the Expanded Composite Cardiovascular Outcome [Time Frame: from randomisation (visit 3; month 0) to last contact (visit 16; up to month 60+30 days)]

Time From Randomisation to First Occurrence of a Composite Microvascular Outcome [Time Frame: from randomisation (visit 3; month 0) to last contact (visit 16; up to month 60+30 days)]

Time From Randomisation to Each Individual Component of the Composite Microvascular Outcome and to the Retinopathy and Nephropathy Composite Outcomes Separately. [Time Frame: from randomisation (visit 3; month 0) to last contact (visit 16; up to month 60+30 days)]

3、SUSTAIN™ 6

Primary Outcome Measures :

Time From Randomisation to First Occurrence of a MACE, Defined as Cardiovascular Death, Non-fatal Myocardial Infarction, or Non-fatal Stroke [Time Frame: Time from randomisation up to end of follow-up (scheduled at week 109)]

Secondary Outcome Measures :

Time From Randomisation to First Occurrence of an Expanded Composite Cardiovascular Outcome [Time Frame: Time from randomisation up to end of follow-up (scheduled at week 109)]

Time From Randomisation to Each Individual Component of the Expanded Composite Cardiovascular Outcome [Time Frame: Time from randomisation up to end of follow-up (scheduled at week 109)]

Time From Randomisation to First Occurrence of All-cause Death, Non-fatal MI, or Non-fatal Stroke [Time Frame: Time from randomisation up to end of follow-up (scheduled at week 109)]

Change From Baseline to Last Assessment in the Trial in Other Treatment Outcomes: Glycosylated Haemoglobin (HbA1c) [Time Frame: Week 0, up to week 104]

Change From Baseline to Last Assessment in the Trial in Other Treatment Outcomes: Fasting Plasma Glucose [Time Frame: Week 0, up to week 104]

Change From Baseline to Last Assessment in the Trial in Other Treatment Outcomes: Body Weight [Time Frame: Week 0, up to week 104]

Change From Baseline to Last Assessment in the Trial in Other Treatment Outcomes: Lipid Profile [Time Frame: Week 0, up to week 104]

Change From Baseline to Last Assessment in the Trial in Other Treatment Outcomes: Urinary Albumin to Creatinine Ratio [Time Frame: Week 0, up to week 104]

Change From Baseline to Last Assessment in the Trial in Other Treatment Outcomes: Vital Signs [Time Frame: Week 0, up to week 104]

Incidence During the Trial in Other Treatment Outcomes: Hypoglycaemic Events [Time Frame: Week 0 - 109]

Incidence During the Trial in Other Treatment Outcomes: Adverse Events [Time Frame: Weeks

0-109]

Occurrence During the Trial in Other Treatment Outcomes: Anti-semaglutide Antibodies [Time Frame: Weeks 0-109]

Change From Baseline to Last Assessment in the Trial in Other Treatment Outcomes: Patient Reported Outcome (PRO) [Time Frame: Week 0, up to week 104]

Change From Baseline to Last Assessment in the Trial in Other Treatment Outcomes: Lipid Profile (Free Fatty Acids) [Time Frame: Week 0, up to week 104]

Change From Baseline to Last Assessment in the Trial in Other Treatment Outcomes: Vital Signs (Pulse Rate) [Time Frame: Week 0, up to week 104]

4、EXSCEL

Primary Outcome Measures :

Primary Efficacy Outcome MACE Events [Time Frame: Time to first event. Information collected during study period (anticipated to be up to 7.5 years).]

Primary Safety Outcome MACE Events [Time Frame: Time to first event. Information collected during study period (anticipated to be up to 7.5 years).]

Secondary Outcome Measures :

Secondary Efficacy Outcome All-Cause Mortality [Time Frame: Time to first event. Information collected during study period (anticipated to be up to 7.5 years).]

Secondary Efficacy Outcome CV Death [Time Frame: Time to first event. Information collected during study period (anticipated to be up to 7.5 years).]

Secondary Efficacy Outcome MI [Time Frame: Time to first event. Information collected during study period (anticipated to be up to 7.5 years).]

Secondary Efficacy Outcome Stroke [Time Frame: Time to first event. Information collected during study period (anticipated to be up to 7.5 years).]

Secondary Efficacy Outcome Hospitalization for ACS [Time Frame: Time to first event. Information collected during study period (anticipated to be up to 7.5 years).]

Secondary Efficacy Outcome Hospitalization for HF [Time Frame: Time to first event. Information collected during study period (anticipated to be up to 7.5 years).]

5、Albiglutide

Primary Outcome Measures :

Time to First Occurrence of Major Adverse Cardiovascular Events (MACE) During Cardiovascular (CV) Follow-up Time Period [Time Frame: Median of 1.65 person years for CV follow-up time period]

Secondary Outcome Measures :

Time to First Occurrence of MACE or Urgent Revascularization for Unstable Angina [Time Frame: Median of 1.65 person years for CV follow-up time period]

Time to Adjudicated CV Death [Time Frame: Median of 1.65 person years for the CV follow-up time period]

Time to First Occurrence of Adjudicated MI [Time Frame: Median of 1.65 person years for CV follow-up time period]

Time to First Occurrence of Adjudicated Stroke [Time Frame: Median of 1.65 person years for CV follow-up time period]

Time to First Occurrence of Adjudicated CV Death or Hospitalization for Heart Failure (HF) [Time Frame: Median of 1.65 person years for CV follow-up time period]

Time to Initiation of Insulin of More Than 3 Months Duration for Those Participants Not Treated With Insulin at Study Start [Time Frame: Up to 2.7 years]

Time to Initiation of Prandial Insulin in Those Participants on Basal Insulin at Study Start [Time Frame: Up to 2.7 years]

Percentage of Participants Achieving Composite Metabolic Endpoint [Time Frame: Months 8, 16, 24 and final assessment (up to 2.7 years)]

Time to First Occurrence of a Clinically Important Microvascular Event [Time Frame: Up to 2.7 years]

Change From Baseline in HbA1c [Time Frame: Baseline and Months 8 and 16]

Change From Baseline in Body Weight [Time Frame: Baseline and Months 8 and 16]

Change From Baseline in Treatment Related Impact Measures-Diabetes (TRIM-D) Total Score [Time Frame: Baseline and Months 8 and 16]

Change From Baseline in EuroQol- 5 Dimension (EQ-5D) Visual Analogue Scale (VAS) Score [Time Frame: Baseline and Months 8 and 16]

Time to Death [Time Frame: Median of 1.73 years for the Vital Status follow-up time period]

Number of Participants With Non-fatal Serious Adverse Events (SAEs) [Time Frame: Up to 2.7 years]

Number of Participants With Adverse Events (AEs) Leading to Discontinuation of Investigational Product (AELD) [Time Frame: Up to 2.7 years]

Number of Participants With AEs of Special Interest [Time Frame: Up to 2.7 years]

Change in Estimated Glomerular Filtration Rate (eGFR) Calculated Using Modification of Diet in Renal Disease (MDRD) Formula [Time Frame: Baseline and Months 8 and 16]

Change From Baseline in Blood Pressure [Time Frame: Baseline and Months 8,16,24 and end of study (up to 2.7 years)]

Change From Baseline in Heart Rate [Time Frame: Baseline and Months 8, 16, 24 and end of study (up to 2.7 years)]

6、REWIND

Primary Outcome Measures :

Number of Participants Who Experienced an Event For Time, From Randomization to First Occurrence of Cardiovascular Death, Non-fatal Myocardial Infarction, or Non-fatal Stroke (a Composite Cardiovascular Outcome) [Time Frame: From randomization to first occurrence or death from any cause or study completion (Median Follow-Up of 5.4 Years)]

Secondary Outcome Measures :

Number of Participants Who Experienced an Event for Time to First Occurrence After Randomization of Cardiovascular Death, Non-fatal Myocardial Infarction, or Non-fatal Stroke, Individually [Time Frame: From randomization to first occurrence or study completion (Median Follow-Up of 5.4 Years)]

Number of Participants Who Experienced an Event for Time to All-cause Mortality [Time Frame: From randomization to study completion (Median Follow-Up of 5.4 Years)]

Number of Participants Who Experienced an Event for Time to First Occurrence After Randomization of the Composite Microvascular Endpoint [Time Frame: From randomization to first occurrence or study completion (Median Follow-Up of 5.4 Years)]

Number of Participants Who Experienced An Event for Time to First Occurrence After Randomization of Heart Failure Requiring Hospitalization or an Urgent Heart Failure Clinic Visit [Time Frame: From randomization to first occurrence or study completion (Median Follow-Up of 5.4 Years)]

Number of Participants Who Experienced an Event for Time to First Occurrence After

Randomization of First Hospitalization for Unstable Angina [Time Frame: From randomization to first occurrence or study completion (Median Follow-Up of 5.4 Years)]

7、PIONEER 6

Primary Outcome Measures :

Time From Randomisation to First Occurrence of a Major Adverse Cardiovascular Event (MACE) Composite Endpoint Consisting of: Cardiovascular Death, Non-fatal Myocardial Infarction or Non-fatal Stroke [Time Frame: Maximum treatment duration is dependent on event rates and is estimated to be no longer than 19 months + 5 weeks of follow-up period.]

Secondary Outcome Measures :

1、Time From Randomisation to First Occurrence of an Expanded Composite Cardiovascular Endpoint Consisting of: Cardiovascular Death, Non-fatal Myocardial Infarction, Non-fatal Stroke, UAP Requiring Hospitalisation or Hospitalisation for Heart Failure [Time Frame: Maximum treatment duration is dependent on event rates and is estimated to be no longer than 19 months + 5 weeks of follow-up period.]

2、Time From Randomisation to First Occurrence of Each of the Individual Components in the Expanded Composite Cardiovascular Endpoint [Time Frame: Maximum treatment duration is dependent on event rates and is estimated to be no longer than 19 months + 5 weeks of follow-up period.]

3、Time From Randomisation to First Occurrence of a Composite Endpoint Consisting of: All-cause Death, Non-fatal Myocardial Infarction or Nonfatal Stroke [Time Frame: Maximum treatment duration is dependent on event rates and is estimated to be no longer than 19 months + 5 weeks of follow-up period.]

4、Time From Randomisation to First Occurrence of Fatal or Non-fatal Myocardial Infarction [Time Frame: Maximum treatment duration is dependent on event rates and is estimated to be no longer than 19 months + 5 weeks of follow-up period.]

5、Time From Randomisation to First Occurrence of Fatal or Non-fatal Stroke [Time Frame: Maximum treatment duration is dependent on event rates and is estimated to be no longer than 19 months + 5 weeks of follow-up period.]

6、Time From Randomisation to All-cause Death [Time Frame: Maximum treatment duration is dependent on event rates and is expected to be no longer than 19 months + 5 weeks of follow-up period.]

7、Time to First AE Leading to Permanent Trial Product Discontinuation [Time Frame: Maximum treatment duration is dependent on event rates and is expected to be no longer than 19 months + 38 days of ascertainment window.]

8、Change in Eye Examination Category [Time Frame: Week -3, End of treatment]

9、Change in Pulse Rate [Time Frame: Week 0, End of treatment]

10、Change in Systolic and Diastolic Blood Pressure [Time Frame: Week 0, End of treatment]

11、Change in Glycosylated Haemoglobin (HbA1c) [Time Frame: Week 0, End of treatment]

12、Change in Body Weight [Time Frame: Week 0, End of treatment]

13、Change in Total Cholesterol - Ratio to Baseline [Time Frame: Week 0, End of treatment]

14、Change in LDL-cholesterol - Ratio to Baseline [Time Frame: Week 0, End of treatment]

15、Change in HDL-cholesterol - Ratio to Baseline [Time Frame: Week 0, End of treatment]

16、Change in Triglycerides - Ratio to Baseline [Time Frame: Week 0, End of treatment]

8、AMPLITUDE-O

Primary Outcome Measures ：

Time to First Occurrence of Major Adverse Cardiovascular Events (MACE): Event Rate Per 100 Participant-years for First Occurrence of Major Cardiovascular (CV) Event - Non-Inferiority Analysis [Time Frame: From Day 1 until the date of first adjudicated and confirmed occurrence of major CV event (maximum duration: up to 31.5 months)]

Secondary Outcome Measures ：

Time to First Occurrence of Major Adverse Cardiovascular Events: Event Rate Per 100 Participant-years for First Occurrence of Major Cardiovascular Event - Superiority Analysis [Time Frame: From Day 1 until the date of first adjudicated and confirmed occurrence of major CV event (maximum duration: up to 31.5 months)]

Time to First Occurrence of the Expanded Major Adverse Cardiovascular Events Composite Events: Event Rate Per 100 Participant-years for First Occurrence of Expanded Major Cardiovascular Event [Time Frame: From Day 1 until the date of first adjudicated and confirmed occurrence of major CV event (maximum duration: up to 31.5 months)]

Time to First Occurrence of Composite Renal Endpoint: Event Rate Per 100 Participant-years for First Occurrence of Composite Renal Endpoint [Time Frame: From Day 1 until the confirmed occurrence of composite renal endpoint (maximum duration: up to 31.5 months)]
