# Supplementary figures and images for: Risk of stroke and retinopathy during GLP-1 receptor agonist cardiovascular outcome trials: An eight RCTs meta-analysis
Source: Front Endocrinol (Lausanne). 2022 Dec 5;13:1007980. doi: 10.3389/fendo.2022.1007980 (PMC9760859; doi:10.3389/fendo.2022.1007980)

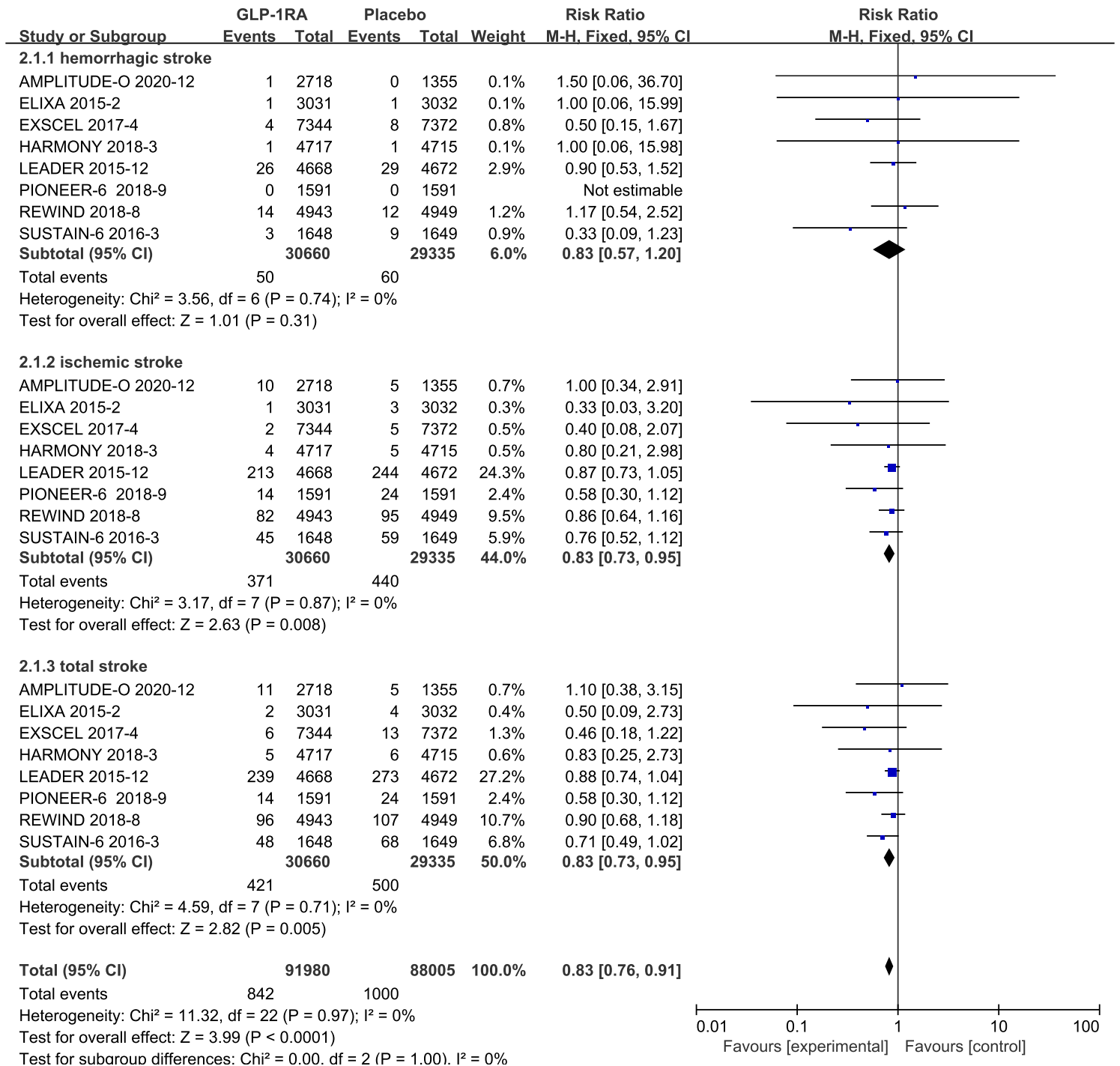

Supplement: Supplementary file 2 [file DataSheet_1.pdf]
